# Review of the genus *Bolbochromus* (Coleoptera, Scarabaeoidea, Geotrupidae, Bolboceratinae) in the Philippines

**DOI:** 10.3897/zookeys.842.32315

**Published:** 2019-05-07

**Authors:** Chun-Lin Li, Jan Krikken, Chuan-Chan Wang

**Affiliations:** 1 The Experimental Forest, National Taiwan University, Chushan, Nantou County 557, Taiwan National Taiwan University Taipei City Taiwan; 2 Naturalis Biodiversity Center/ National Museum of Natural History, 2300 RA Leiden, Netherlands Naturalis Biodiversity Center Leiden Netherlands; 3 Department of Life Science, Fu Jen Catholic University, Hsinchuang, New Taipei City 24205, Taiwan Fu Jen Catholic University New Taipei City Taiwan

**Keywords:** Scarabaeoidea, dor beetles, taxonomy, checklist, key, new species, oriental region

## Abstract

The genus *Bolbochromus* Boucomout, 1909 from the Philippines is reviewed for the first time. Six species in two subgenera, *Metabolbochromus* Krikken & Li, 2013 and *Bolbochromus*, including three new species, Bolbochromus (Bolbochromus) jengi Li & Krikken, **sp. n.**, Bolbochromus (Bolbochromus) luzonensis Li & Krikken, **sp. n.**, and Bolbochromus (Bolbochromus) setosifrons Li & Wang, **sp. n.**, are described with diagnoses, illustrations, distributional data and remarks. A key for the identification of Philippine species is provided. An annotated checklist of the genus in the Philippines is given with information for each species including literature review, synonymy, distribution, and type locality.

## Introduction

*Bolbochromus* Boucomont, 1909 is a group of small to medium-sized (approximately 5.8–13.0 mm body length) bolboceratine beetles which can be distinguished from relatives by having the dorsum shiny, pronotal midline indented, and they are usually bicolored with brownish yellow, reddish brown or dark markings throughout the dorsal surface of the body. The horn-like protrusion on the head and the male genitalia in adults of the genus varies greatly interspecifically and two subgenera, *Bolbochromops* Krikken & Li, 2013 and *Metabolbochromus* Krikken & Li, 2013, were erected for the separation of subgroups within the genus (Krikken and Li 2013).

Unlike other bolboceratine taxa, *Bolbochromus* species are forest dwellers which have a distribution throughout the Oriental region and a small part of the Palaearctic region in East Asia. The number of the known *Bolbochromus* species has been increased in the past decade as a result of discoveries of new taxa housed in museums (Krikken and Li 2013), or by the use of improved collecting methods (e.g. flight intercept trap) conducted in Southeast Asia (Hanski and Krikken 1991; Davis 2000; Li et al. 2008; Li et al. 2013; Krikken and Li 2013). The collecting method of a few recently described species has not been mentioned (Ochi et al. 2010; Ochi et al. 2011; Keith 2012). Currently, there are 26 species known in the genus, including the three new species described here, making it the most diverse bolboceratine genus in Asia.

*Bolbochromus* is the only bolboceratine genus known in the Philippines and has never been systematically reviewed. In fact, the first species in the genus from the Philippines was not described until 2010 (Ochi et al. 2010), and two additional species were named or recorded in 2013 (Krikken and Li 2013). In recent years, a series of *Bolbochromus* specimens has been collected from selected islands of the Philippines by use of flight intercept traps; these collections constitute the basis of this preliminary review of the Philippine fauna. Further investigation in different islands using appropriate collecting methods will undoubtedly increase the number of species in the Philippines as well as provide additional pieces of the puzzle for our knowledge on the diverse *Bolbochromus* fauna in the region.

## Materials and methods

All specimens of the new species described in this paper were collected by flight intercept traps (FIT) and the type specimens were pinned or glued on cards with printed collecting labels for preservation. The habitus images of *Bolbochromus* adults were taken by Canon 7D digital camera. Images of the detailed parts, including the male genitalia, were captured by a Leica M205C stereo microscope equipped with Leica MC190HD microscope camera or by a Hitachi TM3030 plus tabletop Scanning Electron Microscope. The color images were processed using Helicon Focus 6.8.0 to increase the depth of field of an image and all images were edited in Adobe Photoshop 7.0 (background removed, images integrated, numbered and scale bar added). The measurements of specimens, treatment of male genitalia, and external morphological terms used in this paper follow Li et al. (2013).

## Systematics

### Key to *Bolbochromus* species in the Philippines

**Table:** 

1	Horn at apex of clypeus and middle of base of frons well developed; parameres swollen, ovoid capsule-like in shape, fused with a large opening at base	**subgenus Metabolbochromus, *M.catenatus* (Lansberge)**
–	Horn at frons varying in size or with only small conical convexity at center/base of frons, apex of clypeus with or without small convexity; parameres well-separated, as flat as basal piece in lateral view	**subgenus Bolbochromus 2**
2	Eyes small, canthus wide (Figs 19–23); frons glabrous; pronotal midline distinctly indented; punctures on pronotum and elytra coarse	**3**
–	Eyes large, canthus narrow (Fig. 24); frons with 2 long, robust seta laterad of frontal tubercle; punctures on surface of pronotum and elytra fine	***B.setosifrons* Li & Wang, sp. n.**
3	Body longer than 9.0 mm; anterior side of pronotum behind head sharply declivous upright; parameres with a tuft of long setae at dorsal base	**4**
–	Body shorter than 7.2 mm; anterior side of pronotum behind head smoothly declivous; parameres glabrous	**5**
4	Body overall black; frons with a small conical convexity at center; anterior side of pronotum behind head sharply declivous; apex of median lobe of paramere fimbriate	***B.jengi* Li & Krikken, sp. n.**
–	Body brown to blackish brown; frons with a horn at center; anterior side of pronotum behind head upright; apex of median lobe of paramere with a hook-like sclerite	***B.hirokawai* Ochi, Kon & Kawahara**
5	Body dorsum reddish brown to yellowish brown with black markings on head scutellum, and pronotum varying in size or completely absent, markings at center of elytron varying in range across striae 4–10 maximum to striae 8–10 minimum; coarse punctures widely distributed on both sides of pronotum; parameres half length of basal pieces	***B.luzonensis* Li & Krikken, sp. n.**
–	Body dorsum black, pronotum with yellowish brown markings along lateral sides as well as midline or might entirely yellowish brown as shown in Fig. 17, each elytron with three markings, two along suture and one close to umbone; coarse punctures concentrated on disc on both sides of pronotal midline; parameres equal in length to basal pieces	***B.mindanaicus* Krikken & Li**

### Checklist of the genus *Bolbochromus* Boucomont in the Philippines

#### Bolbochromus (Metabolbochromus) catenatus (Lansberge, 1886)

*Bolbocerascatenatus* Lansberge, 1886: 135. Original description.

*Bolbochromuscatenatus*: Boucomont 1909: 117 (new generic combination).

**Distribution.** Sumatra; Borneo; the Philippines (type locality: “Sumatra, Borneo”).

#### Bolbochromus (Bolbochromus) hirokawai Ochi, Kon & Kawahara, 2010

*Bolbochromushirokawai* Ochi, Kon & Kawahara, 2010: 97. Original description.

**Distribution.** Negros, the Philippines (type locality: “Mt. Canlaon”).

#### Bolbochromus (Bolbochromus) jengi Li & Krikken, sp. n.

**Distribution.** Luzon and Babuyan islands, the Philippines (type locality: “Panan, Camiguin Island”).

#### Bolbochromus (Bolbochromus) luzonensis Li & Krikken, sp. n.

**Distribution.**

Luzon, the Philippines (type locality: “Sta. Praxedes Macatel Falls”).

#### Bolbochromus (Bolbochromus) mindanaicus Krikken & Li, 2013

*Bolbochromusmindanaicus* Krikken & Li, 2013: 506. Original combination.

**Distribution.**

Mindanao, the Philippines (type locality: “Dapitan”).

#### Bolbochromus (Bolbochromus) setosifrons Li & Wang, sp. n.

**Distribution.** Leyte, the Philippines (type locality: “Danao Lake”).

### Taxonomy

#### Bolbochromus (Metabolbochromus) catenatus

Taxon classificationAnimaliaColeopteraGeotrupidae

(Lansberge, 1886)

Figs 1 , 2 , 13 , 19 , 20 , 25 , 26 , 33

##### Material examined.

PHILIPPINES: Luzon, Quezon Province, Real, Brgy Cawayan, nr stream, 80m asl, 14°39'56"N, 121°35'35"E, 08-10.IV.2017, by FITs, ML Jeng, H Cahilo (3 females in the collections of Chun-lin Li, CCLI); PHILIPPINES: Palawan Province, Balabac Is., Brgy Malaking Ilog, E coast, 1 km SE to Balabac, near stream, 50m asl, 07°58'45"N, 117°04'23"E, 13–16.VI. 2017, by FITs, ML Jeng, H Cahilog (1 male and 1 female at CCLI); PHILIPPINES. Sibuyan Isl., S-coast, Mabini, Barangay, II-3-III-1999, 0–100 m (Howden collection, the Canadian Museum of Nature, Ottawa).

##### Diagnosis.

**Figures 1–6.**
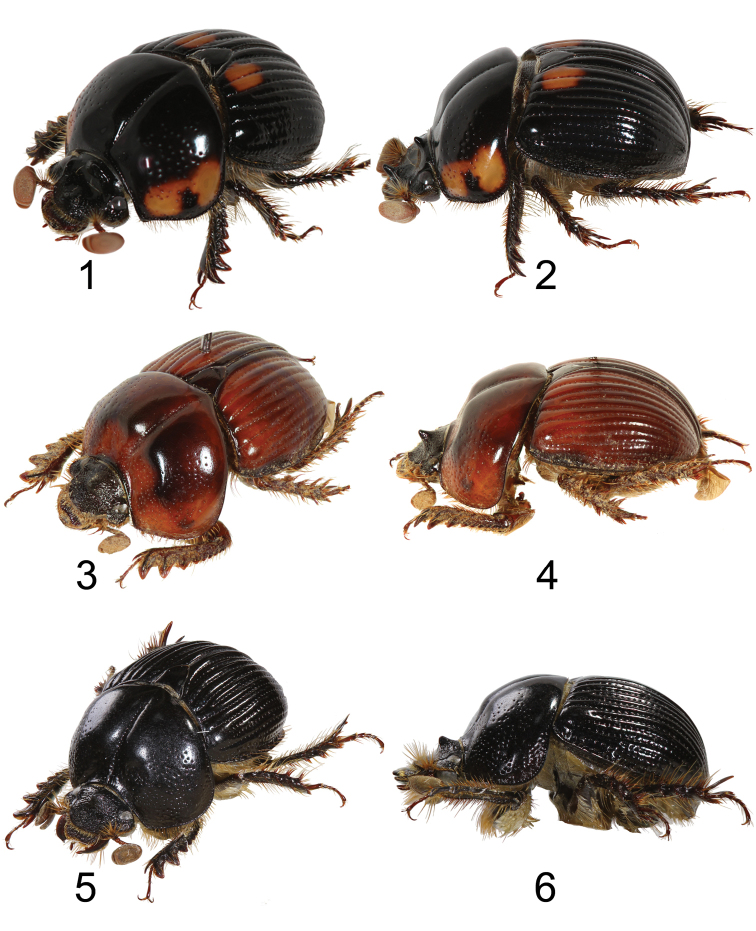
Left oblique view and lateral view of *Bolbochromus* spp. **1–2**B. (Metabolbochromus) catenatus, male **3–4**B. (Bolbochromus) hirokawai, female **5–6**B. (B.) jengi sp. n., holotype male.

Body length 9.4–10.5 mm. Dorsum black to brownish dark with yellowish orange markings along lateral sides of pronotum (markings separated or contiguous, except for fovea) (Figs 1, 2) and across intervals 2–3 at sides of scutellum (Fig. 13); eyes small, canthus wide and simple; horn at middle of anterior margin of clypeus and middle of base of frons well developed; frons glossy, distinctly concave, with 4–5 setigerous punctures, setae long and robust, transversally aligned (Fig. 19, 20); clypeofrontal suture invisible; coarse punctures concentrated on disc of sides of pronotal midline and along lateral margins, anterior side of pronotum behind head upright (female) to sharply declivous (male), face concave, midline deeply indented; each elytron with 7 coarsely punctate striae between suture and humeral umbone, stria 2 interrupted by stria 1 not reaching base, stria 5 terminated in length subequal to stria 2; intervals 1, 3 and 4 more convex and wider than others, interval 2 less convex than others; parameres strongly swollen, ovoid capsule-like in shape, fused with a large opening at base (Figs 25, 26, 33).

##### Distribution.

Philippines (Fig. 39), Borneo, Sumatra.

##### Remarks.

This species is the only representative of the subgenus Metabolbochromus in the Philippines and is also widely distributed from Borneo to Sumatra. Adults are variable in size and the shape of dorsal markings and it was considered by Krikken and Li (2013) that a potential subspecies complex existed. In fact, the pair of specimens collected from Palawan that we treat in this paper have 4 or 5 long, robust setae (Figs 25, 26) on their frons, a feature that has not been observed in other specimens discussed in this paper, which may be due to them having been worn off. For the stability of name usage, we refrain from erecting a new species or subspecies herein and await additional material being available in future.

#### Bolbochromus (Bolbochromus) hirokawai

Taxon classificationAnimaliaColeopteraGeotrupidae

Ochi, Kon & Kawahara, 2010

Figs 3 , 4 , 14

##### Material examined.

Mt. Canlaon Negros Is. PHILIPPINES, XII. 1988 (1 female at National Museum of Nature & Science, Tsukuba, Japan).

##### Diagnosis.

A distinct large *Bolbochromus* species that can be separated from other congeners by the following combination of characters: body length 9.0–10.4 mm; dorsum brown to blackish brown, head, scutellum and elytral suture blackish brown, sides of pronotal midline varied (Fig. 3, 4, 14); eyes small, canthus wide and simple; anterior margin of clypeus beaded with a small convexity at middle and two at side, horn at middle of frons well developed with a small convexity at side of eye, frons overall coarsely punctuate, rugose; clypeofrontal suture vestigial; anterior side of pronotum behind head upright, face concave, coarse punctures concentrated on anterior and lateral sides of pronotum, midline deeply indented, coarsely punctate; scutellum sparsely punctate; each elytron with 7 coarsely punctate striae between suture and humeral umbone, stria 2 interrupted by stria 1 not reaching base, stria 5 terminated in length subequal to stria 2; intervals 1, 3 and 4 more convex and wider than others, interval 2 less convex than others; parameres moderately swollen, a tuft of long setae at dorsal base of parameres, half in length of basal piece; apex of median lobe bilobate with a hook-like sclerite, lateral sclerite well developed.

##### Distribution.

Negros Island, central Philippines (Fig. 39).

##### Remarks.

We have examined the species based on a single female which was collected at the type locality, and this specimen agrees with the original description of the species. *Bolbochromushirokawai* has distinct features such as body color pattern and structures of the head and pronotum that make it easily distinguishable from others within the genus.

**Figures 7–12.**
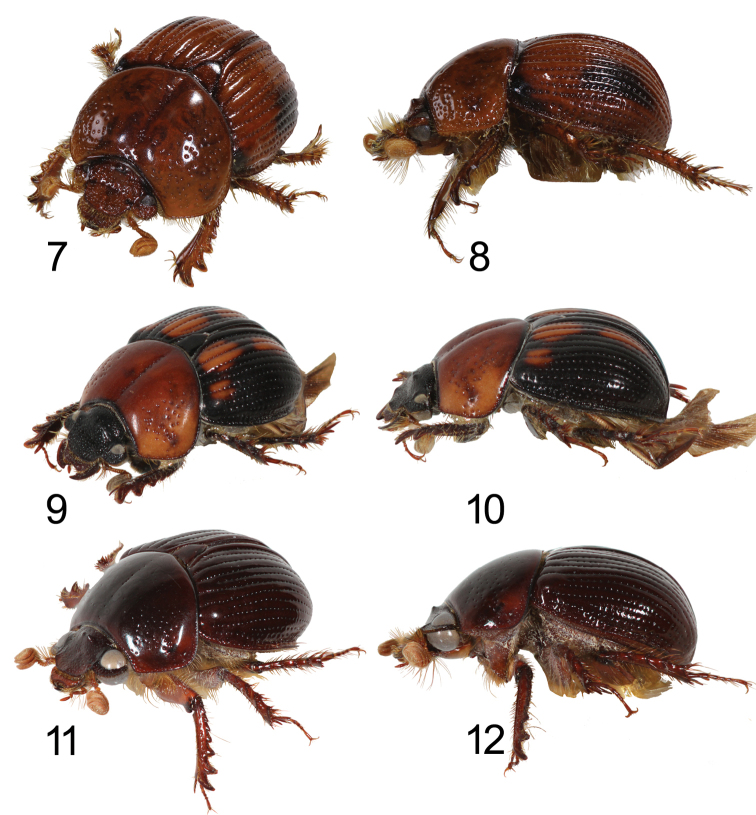
Left oblique view and lateral view of *Bolbochromus* spp. **7–8**B. (B.) luzonensis, sp. n., holotype male **9–10**B. (B.) mindanaicus, female **11–12**B. (B.) setosifrons sp. n., holotype male.

#### Bolbochromus (Bolbochromus) jengi

Taxon classificationAnimaliaColeopteraGeotrupidae

Li & Krikken sp. n.

http://zoobank.org/D39A1720-66E3-4B73-BAB8-1121E385324C

Figs 5 , 6 , 15 , 21 , 27 , 28 , 34

##### Material examined.

**Holotype male.** The holotype (glued on card) with the following information on the label: Philippines: Babuyan Islands, Cagayan Province, Is. Camiguin, Panan, 18-20.IV.2012, FIT, ML Jeng, H. Cahilo leg. The holotype is deposited at the National Museum of Natural Science, Taichung, Taiwan (NMNS). Paratypes: 12 males and 9 females: PHILIPPINES: Luzon, Quezon Prov. Polillos Is., Brgy Tamulaya, Sitio Anibong nr. Stream, 12-14. V. 2013, 3FITs, M.-L. Jeng leg (1 female at NMNS); Philippines: Luzon, Cagayan Province, Sta. Praxedes Macatel Falls, 24-26. IV. 2012, FIT, ML Jeng, H Cahilo leg. (1 male at CCLI); Philippines: Luzon, Cagayan Province, Sta. Ana, Nangramoan, 17-22. IV. 2012, FIT, ML Jeng, H Cahilo leg. (1 female at CCLI); Philippines: Dinagat Is., Dinagat Province, Mncp. Loreto Brgy. Panamauan, mine field near stream, 120m asl, 10°25'49"N, 125°37'42"E, 21-22.III.2017, by FITs, ML Jeng, YT Wang, H Cahilog (1 female at NMNS); Philippines: Luzon, Ilocos Norte Province, Adams, 7km N to Adams, near stream, 240m asl, 18°31'35"N, 120°54'47"E, 03-06.IV.2017, by FITs, ML Jeng, H Cahilog (2 males and 1 female at NMNS, 2 males at CCLI); ditto, 01-03.IV.2017 (1 female at CCLI); Philippines: Luzon, Kalinga Prov., Balbalan Mnp. Mabunol, near stream, 995m asl, 17°29'41"N, 121°12'12"E, 21-24.V.2018, by FITs, ML Jeng, TR Chen, H Cahilog (3 males at CCLI; 1male and 1 female at Universitet Zoologisk Museum, Copenhagen, Denmark; 1male and 1 female at Museum für Naturkunde der Humboldt Universität, Berlin, Germany; 1male and 1 female at the Natural History Museum, London, UK; 1 male and 1 female at Naturhistorisches Museum, Wien, Austria).

##### Type locality.

Panan, Camiguin Island, Babuyan Islands, Cagayan Province, northern Philippines.

##### Description.

**Males** (Figs 5, 6, 15). Body length 9.8–10.3 mm; greatest width 6.0-6.4 mm. Form ovate, sides subparallel. Body overall black with dorsum shiny. Antennal clubs yellowish brown to black. *Head*: Labrum with anterior margin shallowly concave centrally, sides notched, surface coarsely, transversely rugose. Labrum and mandibles visible beyond clypeus when viewed dorsally. Clypeus subtrapezoidal, anterior margin beaded with or without a small convexity at middle, protrusion at basal angle moderately to weakly developed, surface coarsely, transversely rugose. Clypeofrontal suture vaguely indicated. Frons with a small conical convexity at center, tip rounded or weakly bilobed. Eye small, canthus wide and simple (Fig. 21). *Thorax*: Anterior side of pronotum behind head sharply declivous with a shallow fossa either side of midline, midline deeply indented on basal half along with coarse punctures. Surface of pronotum with tiny, secondary punctures sparsely distributed evenly, coarse punctures in fossae and both sides of pronotum, sides of basal half of midline and base of pronotum almost impunctate. Fovea vestigial. Scutellum with scattered secondary punctures, slightly longer than wide medially. *Elytron*: With 7 punctate striae between suture and humeral umbone, punctures coarse, stria 2 interrupted by stria 1 not reaching base, stria 5 terminated in length subequal to stria 2; intervals 1, 3 and 4 more convex and wider than others, interval 2 less convex than others. *Legs*: Protibia with 9 distinct teeth on outer margin, apical 3 teeth protruding, tip of apical tooth sharp and curved outwardly. *Male genitalia*: Parameres one-third as long as basal piece, swollen when viewed laterally, strongly sclerotized at base (Figs 27, 28, 34); surface sparsely to moderately punctate with a tuft of long setae located at dorsal base of each paramere. Median lobe bilobate with dorsal sclerite largely reduced within temones; lateral sclerites elongate with apex fimbriate-like; supporting sclerites absent. Internal sac absent. Temones long, strongly sclerotized. Basal piece with apical portion asymmetrical, dorsal base with a few setiferous punctures.

**Figures 13–16.**
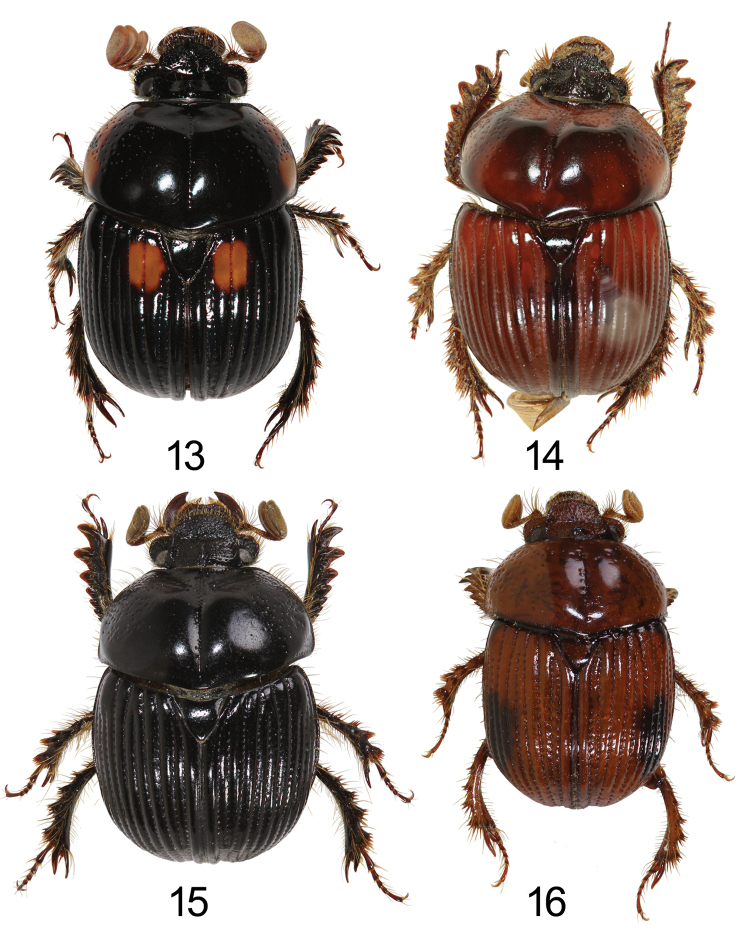
Dorsal habitus of *Bolbochromus* spp. **13**B. (Metabolbochromus) catenatus, male **14**B. (Bolbochromus) hirokawai, female **15**B. (B.) jengi sp. n., holotype male **16**B. (B.) luzonensis sp. n., holotype male.

##### Females.

Similar to male with minor differences of anterior side of pronotum behind head less declivous and fossa in small-sized individual hardly observed, elytral interval between stria 1 and 2 absent, scutellum with one or two coarse punctures, protibia with 10 outer margin teeth and apical tooth of protibia more curved.

##### Diagnosis.

*Bolbochromusjengi* sp. n. is similar to *B.celebensis* Boucomont, 1914 in sharing the overall black body color but it can be distinguished based on the following combination of characters: anterior side of pronotum behind head sharply declivous with a shallow fossa either side of midline (anterior side of pronotum behind head smoothly declivous without fossa at sides of midline in *B.celebensis*); elytral intervals 1, 3 and 4 more convex than others, interval 2 less convex than others (intervals 1–5 equally convex); elytron with 7 striae between suture and humeral umbone (5 striae in *B.celebensis*); elytral striae 2 and 5 not reaching base (all striae reaching base in *B.celebensis*); and male genitalia with a tuft of long setae located at dorsal base of each paramere (glabrous in *B.celebensis*). As the last character above-mentioned on male genitalia of *B.jengi* can also be found in the Negros Island species, *B.hirokawai* indicating their close relationship though the latter species has its dorsal body color yellowish brown to reddish yellow or partly dark brown which can be easily separated from *B.jengi* sp. n.

##### Distribution.

Luzon and neighboring islands, northern Philippines (Fig. 39).

##### Etymology.

*Bolbochromusjengi* sp. n. is named after Dr. Ming-Luen Jeng, the former curator of the National Museum of Natural Science, Taichung, Taiwan, who collected and provided the materials used in this study.

##### Remarks.

*Bolbochromusjengi* sp. n. has its distribution over the neighboring small islands (Map 1) of Luzon and probably also occurs throughout the main island. A noticeable female was collected from Dinagat Island (Map 1), approximately 700 km SE of the nearest known locality of species in Luzon. Further investigation of the species distribution is needed.

**Figures 17–18.**
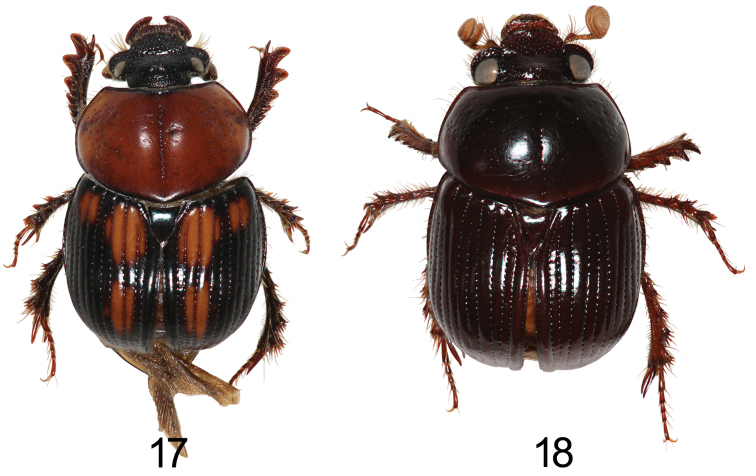
Left oblique view and lateral view of *Bolbochromus* spp. **17**B. (Bolbochromus) mindanaicus, female **18**B. (B.) setosifrons sp. n., holotype male.

#### Bolbochromus (Bolbochromus) luzonensis

Taxon classificationAnimaliaColeopteraGeotrupidae

Li & Krikken sp. n.

http://zoobank.org/1E29AED5-6671-4D38-86C5-639739C3A99B

Figs 7 , 8 , 16 , 22 , 29 , 30 , 35 , 36

##### Material examined.

**Holotype male.** The holotype is glued at a card and with the following information on the label: Philippines: Luzon, Cagayan Province, Sta. Praxedes Macatel Falls, 24-26. IV. 2012, FIT, ML Jeng, H Cahilo leg. The holotype is deposited at the National Museum of Natural Science, Taichung, Taiwan (NMNS). Paratypes: 6 males and 3 females: PHILIPPINES: Luzon, Aurora Province, stream SW of Dinadiawan, 16°02'49"N, 120°42'45"E, 180m asl, 25.V.2016, FIT, ML Jeng, TR Chen, H Cahilo (1 female at CCLI); Philippines: Luzon, Cagayan Province, Sta. Praxedes Macatel Falls, 24-26. IV. 2012, FIT, ML Jeng, H Cahilo leg. (1 male at Museum für Naturkunde der Humboldt Universität, Berlin, Germany; 1male at the Natural History Museum, London, UK; 1 male at NMNS); PHILIPPINES: Luzon, Cagayan Prov. Claveria, 3km SW of Claveria, Nr. mountain top, 80m asl, 18-27. V. 2013, 3FITs, M.-L. Jeng. (1 male at CCLI); Philippines: Luzon, Ilocos Norte Province, Adams, 7 km N of Adams, near stream, 240 m asl, 18°31'35"N, 120°54'47"E, 01-03.IV.2017, by FITs, ML Jeng, H Cahilog (1 male at CCLI); ditto, 03-06.IV.2017 (1 female at CCLI); Philippines: Luzon, Ilocos Norte Province, Adams, Sitio Malaggao, near stream, 370–400m asl, 18°27'13"N, 120°55'10"E, 31.III-02.IV.2017, by FITs, ML Jeng, H Cahilog (1 female at Naturhistorisches Museum, Wien, Austria).

##### Type locality.

Sta. Praxedes Macatel Falls, Cagayan Province, northern Philippines.

##### Description.

**Males** (Figs 7, 8, 16). Body length 6.3–7.2 mm; greatest width 3.4–3.9 mm. Form ovate, sides subparallel. Dorsum reddish brown to yellowish brown, shiny, with or without black markings located on head, eye canthus, base of pronotum, outer sides of elytra and scutellum, markings greatly varying in size among individuals. Antennal clubs yellowish brown. *Head*: Labrum with anterior margin shallowly concave centrally, sides notched, surface coarsely, transversely rugose. Labrum and mandibles visible beyond clypeus when viewed dorsally. Clypeus subtrapezoidal, anterior margin beaded with or without a small convexity at middle, protrusion at basal angle weakly developed, surface coarsely, transversely rugose. Clypeofrontal suture moderately developed. Frons with a small conical convexity at middle of base, tip rounded or weakly bilobed. Eye small, canthus wide, strongly rugose (Fig. 22). Length of antennal clubs shorter than antennal basal segments combined. *Thorax*: Anterior side of pronotum behind head gradually declivous, midline deeply indented on basal half along with coarse punctures. Surface of pronotum with tiny, secondary punctures sparsely but evenly distributed, large coarse punctures in fossae and both sides of pronotum, sides of basal half of midline and base of pronotum almost impunctate. Fovea vestigial. Scutellum with scattered secondary punctures, slightly longer than wide medially. *Elytron*: With 7 punctate striae between suture and humeral umbone, punctures coarse, stria 2 interrupted by stria 1 not reaching base, stria 5 terminated in length subequal to stria 2; intervals 1, 3 and 4 more convex and wider than others, interval 2 less convex than others. *Legs*: Protibia with 9 distinct teeth on outer margin, apical 2 teeth protruding, tip of apical tooth sharp and curved outwardly; metatibia with dorsal apical spur reaching to tip of metatarsomere 2. *Male genitalia*: Parameres half as long as basal piece, weakly sclerotized, glabrous; surface coarsely punctate (Figs 29, 30). Median lobe trilobite (Figs 35, 36), dorsal sclerite most sclerotized, lip-like in shape and strongly curved downwardly; lateral sclerites short, tightly surrounding dorsal sclerite with apex curved inwardly; supporting sclerites absent. Temones long, elongate with apical part strongly sclerotized and in shape of toothbrush-like. Basal piece with apical portion asymmetrical.

**Figures 19–24.**
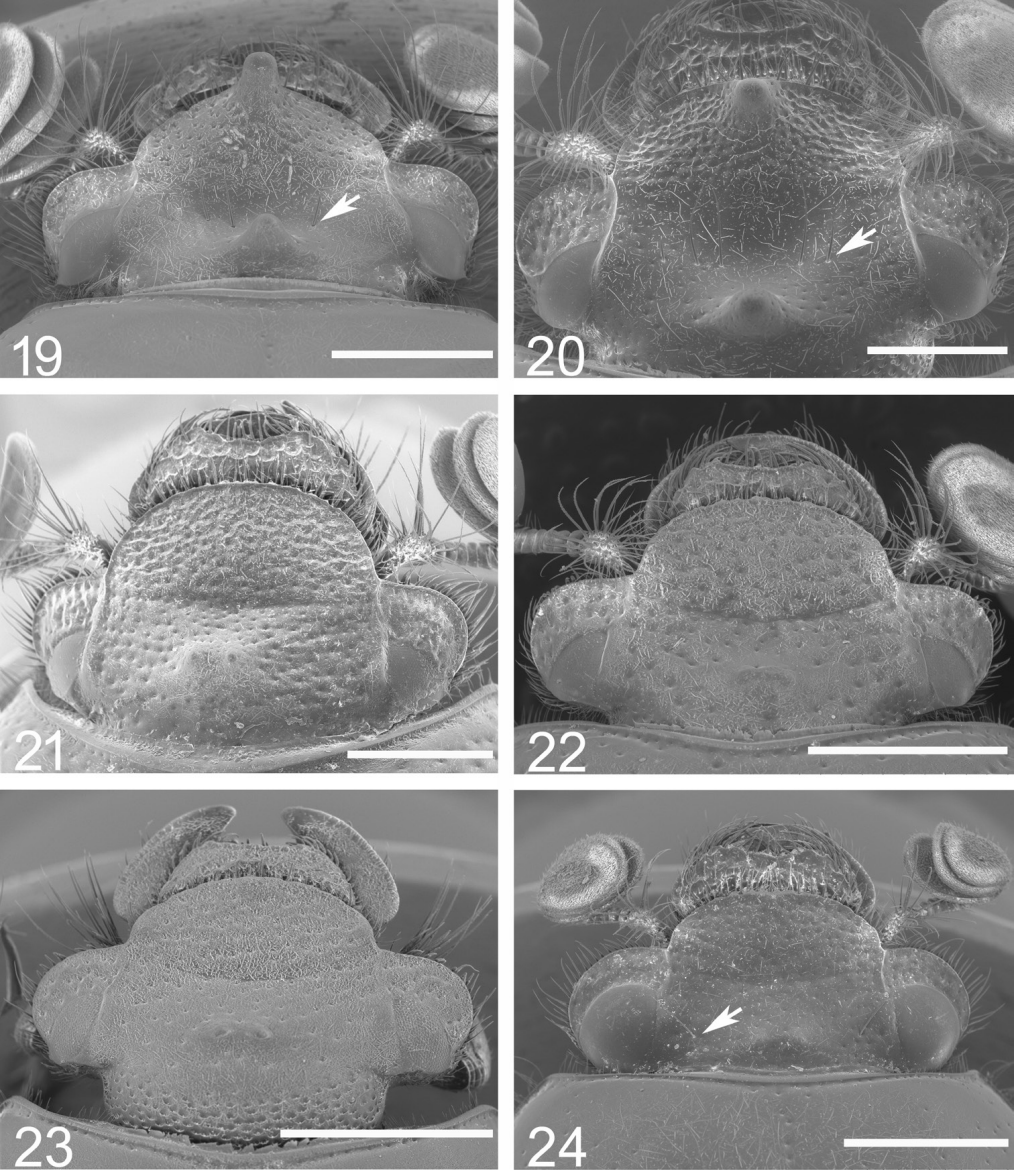
Head of *Bolbochromus* spp. photographed by scanning electron microscope **19**B. (Metabolbochromus) catenatus, male **20**B. (Metabolbochromus) catenatus, female **21**B. (Bolbochromus) jengi, male **22**B. (B.) luzonensis sp. n., male **23**B. (B.) mindanaicus, female **24**B. (B.) setosifrons sp. n., holotype male. Scale bar: 1 mm

##### Females.

Similar to males but minor differences with protibia more robust, protibial outer teeth1 and 2 more broadened and first apical outer teeth more curved.

##### Diagnosis.

*Bolbochromusluzonensis* sp. n. is similar to *B.malayensis* Li & Krikken, 2013 by their smaller body size and dorsal surface markings, but it can be distinguished based on the following combination of characters: length of antennal clubs shorter than antennal basal segments combined (antennal clubs equal in length with antennal basal segments in *B.malayensis*); dorsum reddish brown to yellowish brown (black in *B.malayensis*); surface markings brownish yellow in *B.malayensis*); clypeofrontal suture moderately developed (absent in *B.malayensis*); metatibia with dorsal apical spur reaching to tip of metatarsomere 2 (reaching to tip of metatarsomere 3 in *B.malayensis*).

##### Distribution.

Luzon main island, northern Philippines (Fig. 39).

##### Etymology.

The specific name refers to the geographic origin of the type series, namely Luzon island of the Philippines.

##### Remarks.

The distribution of *Bolbochromusluzonensis* sp. n. may be restricted in the main island of Luzon where the species is sympatric with *Bolbochromusjengi* sp. n. and has never been collected from the neighboring islands.

#### Bolbochromus (Bolbochromus) mindanaicus

Taxon classificationAnimaliaColeopteraGeotrupidae

Krikken & Li, 2013

Figs 9 , 10 , 17 , 23

##### Further material examined.

PHILIPPINES: Mindanao, Sultan Kudarat Province, Culumbio, Dataksaub, Tampakan, Datal-Biaw, VIII.2015, H Cahilo (1 female at CCLI);

##### Diagnosis.

A small *Bolbochromus* species that can be separated from other congeners by the following combination of characters: body length 6.0–7.0 mm; dorsum black, pronotum with yellowish brown markings along lateral sides as well as midline or sometimes becoming entirely yellowish brown as in the individual shown in Figs 9, 10, 17; each elytron with three markings, two along suture and one close to umbone, size of markings varied; eyes small, canthus wide and simple; anterior margin of clypeus beaded with a small convexity at middle and two at side, frons with a small convexity at middle of base, tip bilobed, frons overall coarsely punctate (Fig. 23); clypeofrontal suture visible; coarse punctures concentrated on disc of both sides of pronotal midline, punctate midline distinctly indented, punctures coarse; anterior side of pronotum behind head smoothly declivous, coarse punctures concentrated on anterior face and sides of pronotum; scutellum sparsely punctate, punctures small; each elytron with 7 coarsely punctate striae between suture and humeral umbone, stria 2 interrupted by stria 1 not reaching base, stria 5 terminated in length subequal to stria 2; intervals 1, 3 and 4 more convex and wider than others, interval 2 less convex than others; parameres elongate, tips rounded, equal in length of basal pieces; median lobe distinctly sclerotized, without protruding sclerite.

##### Distribution.

Mindanao island, southern Philippines (Fig. 39).

##### Remarks.

The pronotal marking pattern of this species greatly varied where the black portion might be entirely replaced by a yellowish-brown color. On the other hand, elytral markings are variable in size. *Bolbochromusmindanaicus* is widely distributed throughout Mindanao and neighboring islands (e.g. Jolo island roughly 110 km southwest of Mindanao).

**Figures 25–32.**
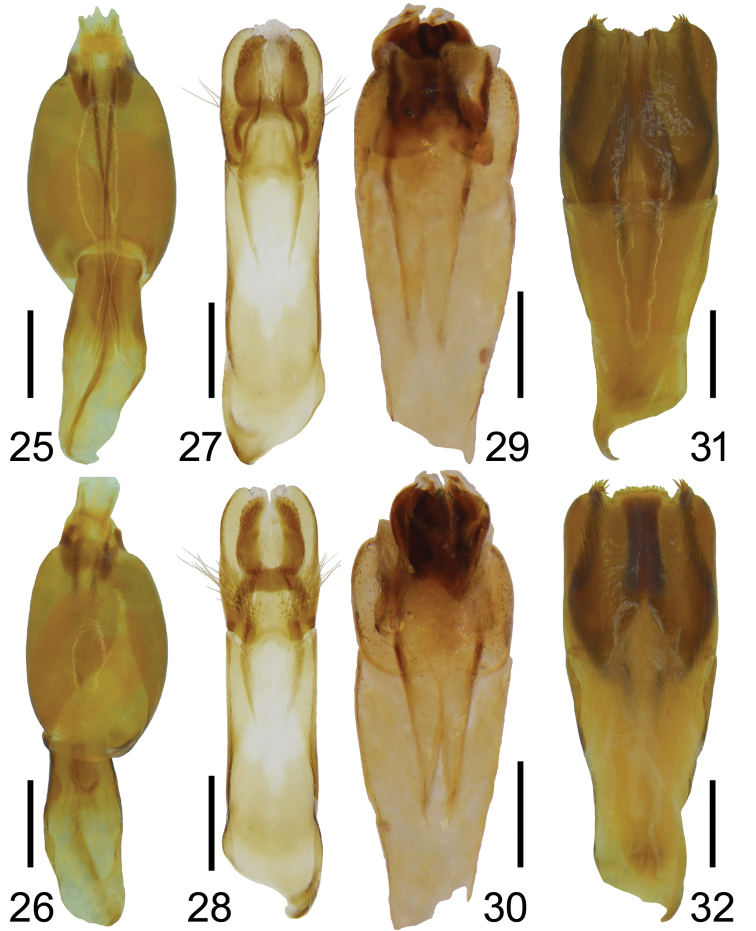
Male genitalia of *Bolbochromus* spp. (**25, 27, 29, 31**, dorsal view; **26, 28, 30, 32**, lateral view) **25–26**B. (Metabolbochromus) catenatus**27–28**B. (Bolbochromus) jengi sp. n. **29– 30**B. (B.) luzonensis sp. n. **31–32**B. (B.) setosifrons sp. n. Scale bar: 0.5 mm.

#### Bolbochromus (Bolbochromus) setosifrons

Taxon classificationAnimaliaColeopteraGeotrupidae

Li & Wang sp. n.

http://zoobank.org/AAE5D53E-E27D-4035-A423-6EC5A268D22E

Figs 11 , 12 , 18 , 24 , 31 , 32 , 37 , 38

##### Material examined.

**Holotype male.** The holotype is glued at a card and with the following information on the label: Philippines: Leyte Is., Leyte Province, Ormoc City, Danao Lake, near stream, 600–700m asl, 11°3'38"N, 124°42'14"E, 9-11. III. 2017, by FITs, ML Jeng, H Cahilog. The holotype is deposited at the National Museum of Natural Science, Taichung, Taiwan (NMNS). Paratype: same data as the holotype (1 male at NMNS).

##### Type locality.

Danao Lake (11°3'38"N, 124°42'14"E), Ormoc city, Leyte Province, Philippines.

##### Description.

**Males** (Figs 11, 12, 18). Body length 7.3–7.7 mm; greatest width 4.7–4.9 mm. Form ovate, sides subparallel. Dorsum blackish brown with labrum brownish orange, shiny, lateral sides and basal margin of pronotum brownish orange in unshaped form. Antennal clubs yellowish brown. *Head*: Labrum with anterior margin shallowly concave centrally, sides notched, surface coarsely, transversely rugose. Labrum and mandibles visible beyond clypeus when viewed dorsally. Clypeus subtrapezoidal (Fig. 18), anterior margin beaded with a small convexity at middle, protrusion at basal angle weakly developed, surface moderately punctate. Clypeofrontal suture moderately developed. Frons with a small conical convexity at middle of base, tip weakly bilobed, a long, robust seta located between eye and tubercle. Eye large, canthus narrow and simple. Length of antennal clubs shorter than antennal basal segments combined. *Thorax*: Anterior side of pronotum behind head smoothly declivous, midline shallowly indented along with fine punctures. Surface of pronotum with secondary punctures barely observed, large punctures in fossae and both sides of pronotum sparsely distributed, sides of midline and base of pronotum almost impunctate. Fovea vestigial. Scutellum with scattered secondary punctures, slightly longer than wide medially. *Elytron*: With 7 punctate striae between suture and humeral umbone, punctures fine, stria 2 interrupted by stria 1 not reaching base, stria 5 terminated in length subequal to stria 2; intervals 1, 3 and 4 more convex and wider than others, interval 2 less convex than others. *Legs*: Protibia with 9 distinct teeth on outer margin, apical 2 teeth protruding, tip of apical tooth sharp and curved outwardly; metatibia with dorsal apical spur reaching to tip of metatarsomere 2. *Male genitalia*: Parameres elongate, half as long as basal piece, outer margins well sclerotized; surface sparsely finely punctate (Figs 31, 32). Median lobe trilobite, apex of dorsal sclerite notched; lateral sclerites elongate, longer than dorsal sclerite; apices of dorsal and lateral sclerites tufted with robust, short setae (Figs 37, 38); supporting sclerites well developed. Internal sac invisible. Temones long, swollen and strongly sclerotized at apical one-fourth. Basal piece with apical portion asymmetrical, surface glabrous.

##### Female.

Unknown.

##### Diagnosis.

*Bolbochromussetosifrons* sp. n. can be separated from other known *Bolbochromus* species in the Philippines based on the following combination of characters: eyes larger with narrow canthus; each side of frontal tubercle with a long, robust seta; pronotal midline shallowly indented; punctures on surface of pronotum and elytra fine; apex of genital median lobe tufted with robust, densely short setae.

##### Distribution.

Leyte Island, central Philippines (Fig. 39).

##### Etymology.

The species epithet is a combination of two Latin words: *setosus* (setose) and *frons* (frons). It is named after the long, robust setae which are located on the frons of specimens.

##### Remarks.

*Bolbochromussetosifrons* sp. n. is the first species of the genus recorded from Leyte island and its distinct morphological features (e.g. larger eyes, overall small dorsal punctures and shallow pronotal midline etc.) separate it from other Philippine species.

**Figures 33–38.**
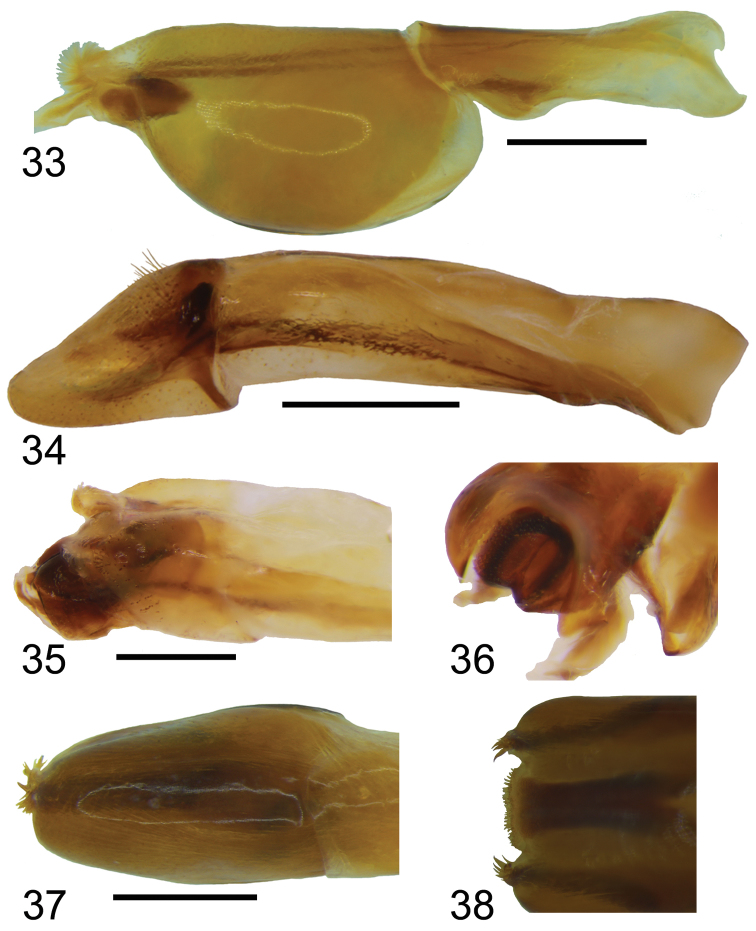
Male genitalia of *Bolbochromus* spp. in lateral view **33**B. (Metabolbochromus) catenatus**34**B. (Bolbochromus) jengi sp. n. **35–36**B. (B.) luzonensis sp. n. **36** tip of median lobe **37– 38**B. (B.) setosifrons sp. n. **38** tip of parameres. Scale bar: 0.5 mm.

**Figure 39.**
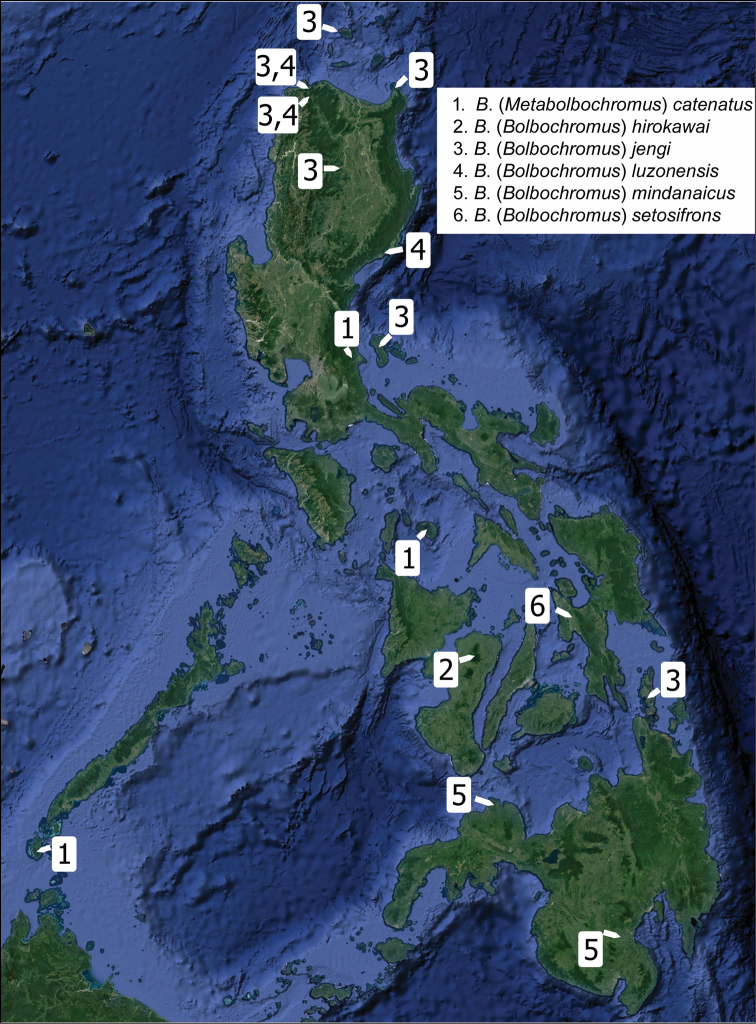
Distribution map of the Philippine *Bolbochromus* species.

## Supplementary Material

XML Treatment for Bolbochromus (Metabolbochromus) catenatus

XML Treatment for Bolbochromus (Bolbochromus) hirokawai

XML Treatment for Bolbochromus (Bolbochromus) jengi

XML Treatment for Bolbochromus (Bolbochromus) luzonensis

XML Treatment for Bolbochromus (Bolbochromus) mindanaicus

XML Treatment for Bolbochromus (Bolbochromus) setosifrons
